# Experimental Persistent Infection of BALB/c Mice with Small-Colony Variants of *Burkholderia pseudomallei* Leads to Concurrent Upregulation of PD-1 on T Cells and Skewed Th1 and Th17 Responses

**DOI:** 10.1371/journal.pntd.0004503

**Published:** 2016-03-14

**Authors:** Jia-Xiang See, Chandramathi Samudi, Alireza Saeidi, Nivedita Menon, Leang-Chung Choh, Jamuna Vadivelu, Esaki M. Shankar

**Affiliations:** 1 Department of Medical Microbiology, Faculty of Medicine, University of Malaya, Lembah Pantai, Kuala Lumpur, Malaysia; 2 Tropical Infectious Disease Research and Education Center (TIDREC), University of Malaya, Lembah Pantai, Kuala Lumpur, Malaysia; 3 Centre of Excellence for Research in AIDS (CERiA), Wisma R & D, University of Malaya, Lembah Pantai, Kuala Lumpur, Malaysia; Fondation Raoul Follereau, FRANCE

## Abstract

**Background:**

*Burkholderia pseudomallei* (*B*. *pseudomallei*), the causative agent of melioidosis, is a deadly pathogen endemic across parts of tropical South East Asia and Northern Australia. *B*. *pseudomallei* can remain latent within the intracellular compartment of the host cell over prolonged periods of time, and cause persistent disease leading to treatment difficulties. Understanding the immunological mechanisms behind persistent infection can result in improved treatment strategies in clinical melioidosis.

**Methods:**

Ten-day LD_50_ was determined for the small-colony variant (SCV) and its parental wild-type (WT) via intranasal route in experimental BALB/c mice. Persistent *B*. *pseudomallei* infection was generated by administrating sub-lethal dose of the two strains based on previously determined LD_50_. After two months, peripheral blood mononuclear cells (PBMCs) and plasma were obtained to investigate host immune responses against persistent *B*. *pseudomallei* infection. Lungs, livers, and spleens were harvested and bacterial loads in these organs were determined.

**Results:**

Based on the ten-day LD_50_, the SCV was ~20-fold less virulent than the WT. The SCV caused higher bacterial loads in spleens compared to its WT counterparts with persistent *B*. *pseudomallei* infection. We found that the CD4+ T-cell frequencies were decreased, and the expressions of PD-1, but not CTLA-4 were significantly increased on the CD4+ and CD8+ T cells of these mice. Notably, persistent infection with the SCV led to significantly higher levels of PD-1 than the WT *B*. *pseudomallei*. Plasma IFN-γ, IL-6, and IL-17A levels were elevated only in SCV-infected mice. In addition, skewed plasma Th1 and Th17 responses were observed in SCV-infected mice relative to WT-infected and uninfected mice.

**Conclusion:**

*B*. *pseudomallei* appears to upregulate the expression of PD-1 on T cells to evade host immune responses, which likely facilitates bacterial persistence in the host. SCVs cause distinct pathology and immune responses in the host as compared to WT *B*. *pseudomallei*.

## Author Summary

Melioidosis is an endemic tropical disease in South East Asia and Northern Australia, which is caused by *Burkholderia pseudomallei*, an environmental bacterium found in the soils of paddy fields and muddy waters across these regions. The bacterium is known to reside within the host cell for prolonged periods of time and is capable of causing long-lasting disease. Recurrent disease is common even with appropriate antibiotic treatments. The mechanisms behind the persistence of *B*. *pseudomallei* in the host are still unclear. We investigated the host cell-mediated immune responses against persistent *B*. *pseudomallei* infection in BALB/c mice. We found a reduced CD4+ T-cell frequency in mice with persistent *B*. *pseudomallei* infection, suggestive of the key role of these cells in experimental melioidosis. Moreover, we also observed significant upregulation of PD-1 on both CD4+ and CD8+ T cells in mice with persistent *B*. *pseudomallei* infection, possibly indicating that the T cells were undergoing exhaustion. Based on our results, we postulated that *B*. *pseudomallei* is able to impair host immune responses, likely by facilitating the depletion of CD4+ T cells and upregulation of PD-1 on T cells, which potentially facilitates bacterial persistence in the host. Targeting T-cell responses could be an approach to develop vaccines or therapeutics against persistent *B*. *pseudomallei* infection.

## Introduction

*Burkholderia pseudomallei* (*B*. *pseudomallei*) is a Gram-negative, aerobic, rod-shaped bacillus that causes melioidosis, a potentially fatal disease endemic across parts of tropical South East Asia and Northern Australia afflicting human and animals[1,2]. *B*. *pseudomallei* has been classified as a category B biothreat agent by the Center for Disease Control and Prevention[3]. The most common routes of infections include percutaneous inoculation, inhalation, and ingestion of contaminated soil, dust, or aerosol[3]. Besides acute infection, it has also been well documented that *B*. *pseudomallei* can cause persistent disease, where the host normally shows little to no signs of infection during the prolonged period of latency[4–6]. Eighty percent of the individuals diagnosed with melioidosis have one or more underlying conditions, suggesting the possibility of persistence of *B*. *pseudomallei* in the host during a prior exposure or infection, and only relapse when the host immunity wanes[3].

Evidence suggests that persistent infections could be associated with small-colony variants (SCVs) [7–11]. SCVs are reportedly defective in growth and form pin-point colonies on agar medium after 24–72 hours [12–14]. Although described in various bacteria, SCVs of *Staphylococcus aureus* are the most extensively studied[14]. SCVs are relatively less susceptible to antibiotics and difficult to treat, causing recurrent diseases [7,8,14,15]. More importantly, SCVs of *B*. *pseudomallei* reportedly have higher degree of antibiotic resistance and distinct virulence-associated proteins [16–18]. Thus, SCVs might be important in melioidosis as relapse after treatment is common [19]. To date, there is only limited *in vivo* information regarding interactions between SCVs and WT and the host.

The adaptive immune response attributes to melioidosis in the host still remains poorly understood. A recent study showed that strong CD4+ and CD8+ T-cell responses were required for patients to survive acute melioidosis[20]. CD4+ T cells were also reported to protect mouse in the late stage of *B*. *pseudomallei* infection [21]. Therefore, robust T-cell functions are paramount to protection against *B*. *pseudomallei* infection in the host. Nevertheless, T-cell responses could likely undergo attrition following increased expression of co-inhibitory molecules on T cells. For instance, the expressions of programmed death-1(PD-1) and cytotoxic T-lymphocyte-associated protein 4(CTLA-4) can cause T-cell exhaustion following engagement with their cognate ligands expressed on host cells [22–25]. Mounting evidence suggests that pathogens, such as *Mycobacterium* spp. and human immunodeficiency virus (HIV), can upregulate PD-1 and CTLA-4 expressions during chronic infections to evade adequate host immune responses[26–31]. A recent study demonstrated that PD-L1 on *B*. *pseudomallei*-infected human polymononuclear neutrophils inhibit T cell activities *in vitro* [32]. However, direct effects of persistent *B*. *pseudomallei* infection on PD-1 expression on T cells using an *in vivo* model have yet to be investigated. Here, we hypothesized that *B*. *pseudomallei* can employ the strategy of upregulating co-inhibitory molecules to evade host immune surveillance, potentially rendering its persistence in the host. To elucidate the role of PD-1 and CTLA-4, we generated a persistent murine model of *B*. *pseudomallei* infection and compared between wild-type and SCVs in regard to the host immune responses. Besides, we also determined Th1, Th2, and Th17 cytokine levels in peripheral blood to understand the immunoregulatory system during persistent *B*. *pseudomallei* infection.

## Materials and Methods

### Ethics Statement

All mouse experiments were performed following the guidelines of the University of Malaya Animal Care and Use Policy (UM ACUP) and the protocols were reviewed and approved by the Animal Experimental Unit of University of Malaya, Kuala Lumpur, Malaysia (Ref. No.: 2014-08-05/MMB/R/JSV). The University of Malaya Animal Care and Use Policy (UM ACUP) is accredited by the Association for Assessment and Accreditation of Laboratory Animal Care (AAALAC), and conforms to all government laws and regulations. It provides for approved research and teaching activities, and safeguards the health and welfare of staff and students involved in scholarly activities using animals or animal parts derived from animals. Animals were maintained with controlled temperature, 12h light /dark cycles and given water and feed *ad libitum*. All efforts were made in order to minimize animal suffering.

### Bacterial Identification

*B*. *pseudomallei* strains OB (WT, INSDC: APLK00000000.1) and OS (SCV, INSDC: APLL00000000.1) were originally isolated as previously described [33] from a clinical melioidosis case admitted to the University Malaya Medical Center (UMMC). OS was differentiated from OB by its slow growth rate on nutrient agar under aerobic conditions. OB produced clear visible colonies within 24 hours, while OS produced small visible colonies within 48 hours. In addition, OB and OS were characterized with commercial analytical profile index API 20NE (Biomeriux) test and *in house* PCR assay specific to *B*. *pseudomallei* [34].

### Growth Curve

A single colony was chosen from OB and OS streaked on nutrient agar, and inoculated into LB broth at 37°C in an incubator with 200rpm. Overnight cultures were diluted with Luria-Bertani (LB) broth. Fifty milliliters of initial cultures with an OD of 600nm (OD_600_) of 0.05 were incubated at 37°C with 200rpm for 48 hours in 250mL conical flasks. OD_600_ of the cultures were measured from time to time. Appropriate dilutions were conducted with LB broth when necessary to obtain readable range of a spectrophotometer.

### Bacterial Inoculum

A single colony from nutrient agar was cultured in Luria-Bertani (LB) broth and incubated overnight with the same conditions as in growth curve. Later, cultures were adjusted to an OD_600_ of 0.05 and incubated again. Cultures that reached the mid-logarithmic phase (OD_600_ 0.5–0.7) were harvested, washed, and re-suspended in phosphate-buffered saline (PBS). Subsequently, the bacterial suspensions were serially diluted ten-fold with PBS until the desired of inoculum was obtained. Inoculum was plated on nutrient agar to enumerate colony- forming unit (CFU).

### Lethal Dose Fifty Percent (LD_50_)

All procedures involving animal infections were performed according to ethics code approved by the Animal Experimental Unit (AAALAC accredited) of University of Malaya. (Ref. No.: 2014-08-05/MMB/R/JSV). Seven to eight-week-old female BALB/c mice were used for infection. They were obtained from University Putra Malaysia, and acclimatized for two weeks prior to infection. Mice were under *ad libitum* feeding conditions in all experiments. Mice were anaesthetized with isoflurane (Piramal Healthcare Ltd), and 10μL of bacterial inoculum was administered via intranasal route. Groups of six mice were infected with different doses, ranging from 10^2^–10^7^ CFU/mouse. Mice were observed daily. Ten days LD_50_ was determined using Reed and Muench method[35].

### Persistent Infection

Persistent infection was generated in mice as described before[36] with slight modifications. Sublethal bacterial dose (~2–8% of LD_50_) was optimized based on previously determined LD_50_ of each strain. Mice were infected with OB and OS strains, respectively. Only mice that survived for ≥60 days after infection were sacrificed for use in the downstream experiments. Mice inoculated with PBS were used as controls, and will be referred as uninfected mice for simplicity.

### Peripheral Blood Mononuclear Cells and Plasma

Mice with persistent *B*. *pseudomallei* infection were anaesthetized with isoflurane, and blood was drawn via terminal cardiac puncture. Heparinized blood samples were centrifuged, and plasma samples were collected and stored at -80°C until use. PBMCs were isolated as described before [37,38]. Briefly, PBMCs were prepared by density-gradient centrifugation over Ficoll-Paque (Sigma Aldrich), and washed twice with PBS. Cell viability was determined by 0.4% trypan blue (Life Technologies) staining.

### Extraction of Viscera

Lungs, livers, and spleens of mice with persistent *B*. *pseudomallei* infection were aseptically harvested and homogenized in PBS. Later, the suspension was ten-fold serially diluted and inoculated on selective Ashdown’s agar[39] at 37°C to examine the presence of *B*. *pseudomallei*. CFU in each organ was enumerated after 48 hours of incubation to determine the bacterial burden in each organ.

### Polymerase Chain Reaction (PCR)

To confirm if *B*. *pseudomallei* grew on Ashdown’s agar, DNA was extracted from the colonies by boiling method, and PCR targeting *B*. *pseudomallei*-specific 16S rRNA gene was performed as described before[40] with slight modifications. The total volume for PCR was 25μL, including 1μL of DNA, 0.2μM of BS3L and BS4R primers each, 5X PCR Master Mix (containing 7.5mM MgCl_2_, 1mM deoxynucloside triphosphates, 10% glycerol, 0,1 unit/μL Taq Polymerase)(i-DNA Biotechnology, Malaysia), and dH_2_O. PCR products were resolved on 1.5% (w/v) agarose gel.

### Polychromatic Flow Cytometry

In each tube, 1x10^6 PBMCs were stained with Fixable Viability Stain 510 (BD Biosciences, cloneR35-95), Alexa Fluor 488 hamster anti-mouse CD3e (BD Biosciences, clone 145-2C11), Pe-Cy7 rat anti-mouse CD4 (BD Biosciences, clone GK1.5), APC-H7 rat anti-mouse CD8a (BD Biosciences, clone 53–6.7), APC hamster anti-mouse PD-1 (BD Biosciences, clone J43), and PE hamster anti-mouse CTLA (BD Biosciences, clone UC10-4F10-11). Respective isotype control for each antibody was used to place proper gate for backgrounds. All antibodies were pre-titrated for optimal working concentration. Data were acquired on 8-color FACS Canto II (BD Biosciences) immunocytometry system with FACS Diva software (BD Bioscience). Data were analyzed using Flowjo software version 10 (Tree Star, Oregon, USA).

### Cytometric Bead Array

Plasma samples collected were used for Cytometric Bead Array (CBA) Mouse Th1/Th2/Th17 Cytokine Kit (BD Biosciences) to measure the levels of IL-2, IL-4, IL-6, IL-10, IL-17A, TNF-α, and IFN-γ according to the manufacturer’s instructions. All results were analyzed using FCAP Array 1.0 (Softflow, USA).

### Statistical Analysis

Two-tailed Mann Whitney-U test was used to determine statistical significance among different groups, due to the assumption that samples might not follow Gaussian distribution. Results were illustrated using Box-Whisker Plots. Multiple correlation analysis was done using a two-tailed Spearman’s Rank Order Correlation. All statistical analysis was done using GraphPad Prism 6 software (La Jolla, USA). Level of significance was first set at *P<0.05, ** *P*<0.01, ***P<0.001, and adjusted with appropriate Bonferroni correction.

## Results

### Growth Rates of WT *B*. *pseudomallei* were Rapid and Attained Higher Growth Densities Compared to SCV

**Fig 1:**
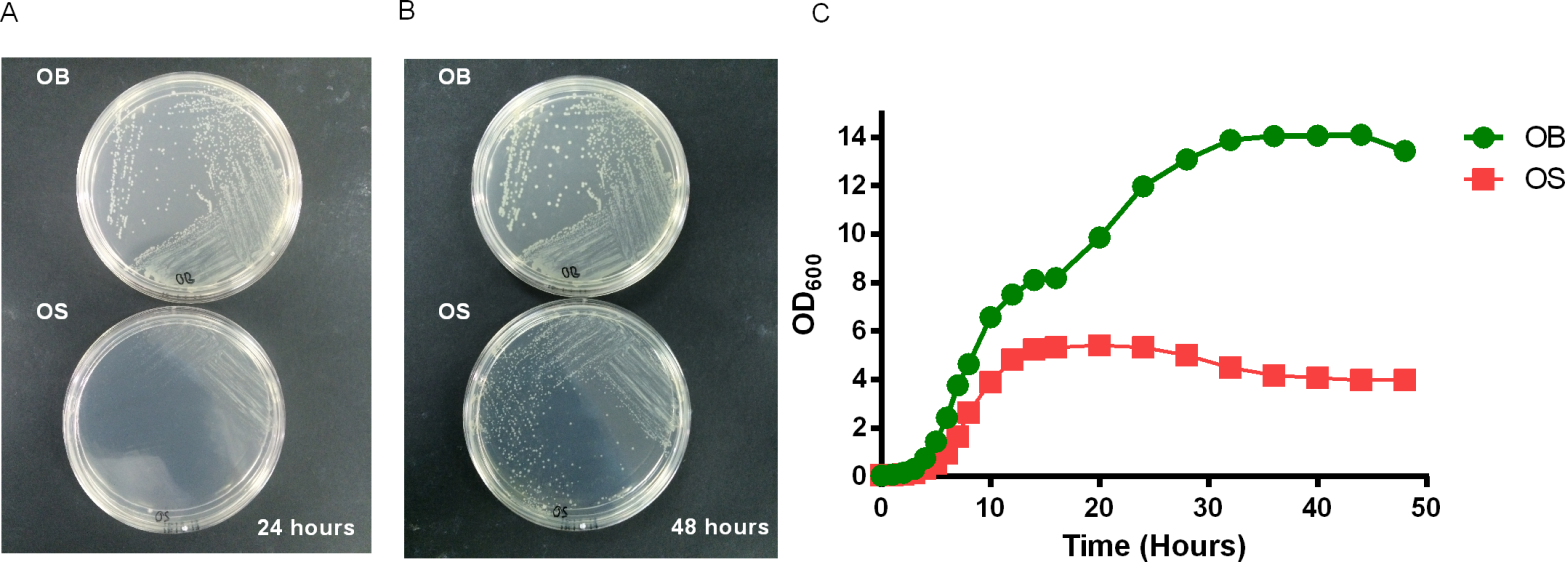
Growth kinetics of OB and OS morphotypes of *B*. *pseudomallei* in *vitro*. Morphology of OB (WT) and OS (SCV) growth on nutrient agar after (A) 24 hours and (B) 48 hours. (C) Growth curves of OB and OS in LB broth incubated at 37°C for 48 hours. Data are representative of at least 2 independent experiments (A-C).

We first compared the growth kinetics of OB (WT) and OS (SCV) to characterize phenotypic differences observed after aerobic incubation on nutrient agar at 37°C (Fig 1A and 1B). Our growth kinetics results revealed distinct growth profiles between the two morphotypes. We found that the growth of OB was relatively rapid as compared to the OS morphotype. OB showed a very short lag phase, and it doubled its OD_600_ reading within the first hour (Fig 1C) of growth. On the other hand, OS showed a lag phase of ~2 hours, and it started to double its OD_600_ reading from the third hour onwards. After entering the log phase, OB reached its stationary phase after 32 hours, with the OD_600_ reading reaching 14. OS reached its stationary phase after 14 hours, which was much earlier than OB, with the OD_600_ reading only reaching 5.5. OB did not reach its death phase within 48 hours, while OS reached its death phase after 28 hours. Our results indicated a defect in SCV to grow *in vitro*, compared to the WT morphotype of *B*. *pseudomallei*.

### SCVs were Relatively Less Virulent than WT *B*. *pseudomallei* in BALB/c Mice

**Fig 2:**
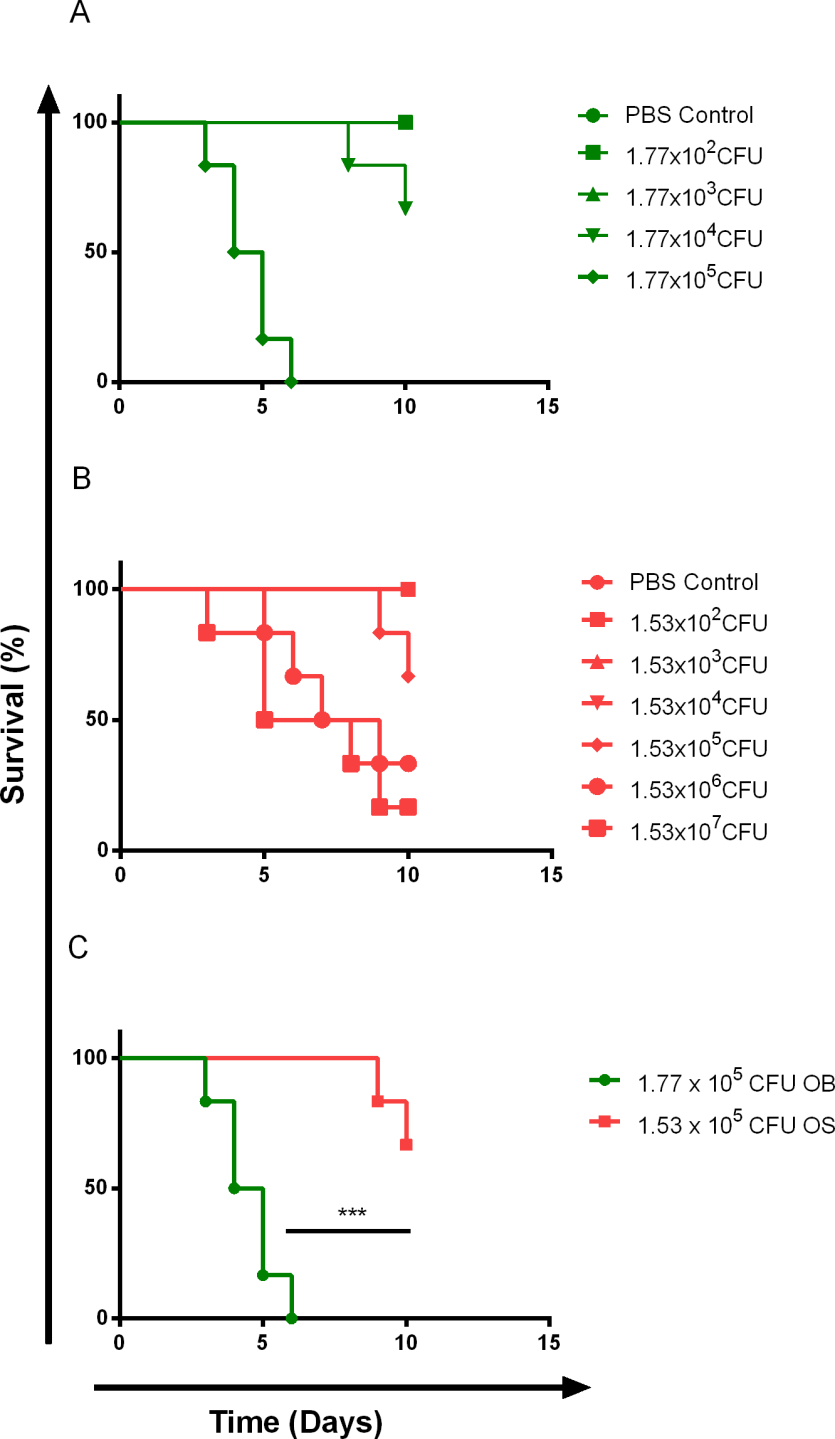
Ten-day survival rate of BALB/c mice infected with WT and SCV *B*. *pseudomallei*. BALB/c mice were infected with different doses of (A) OB and (B) OS, and ten-day survival rate of mice was plotted. (C) Comparison of ten-day survival rate of mice using same log CFU of OB and OS, and *P* value was calculated using Log-Rank test. *P<0.05, **P<0.01, ***P<0.001. Data are representative from 2 independent experiments (A-C, n = 6 per bacterial dose).

Next, we seek to understand the virulence of WT and SCV *in vivo*, in the interest of determining appropriate sub-lethal dose for persistent *B*. *pseudomallei* infection. We compared the virulence of OB and OS using a BALB/c mouse model. Ten days survival rate of BALB/c mice in each group infected with OS and OB via intranasal route was observed (Fig 2A and 2B). We noticed that 1.77x10^5^ CFU of OB was able to kill all animals within 7 days, while a 10-fold lower dose of OB was only capable of killing two in 10 days. We observed that OS was less virulent than OB, with 1.53x10^5^ CFU of OS only killed two in 10 days and 1.53x10^6^ CFU of OS killed four in 8 days. In addition, we performed Log-Rank test to investigate the survival curves between same log CFU of OB and OS, and confirmed that OS was significantly less virulent than OB (*P*<0.001; Fig 2C). Ten days LD_50_ was calculated using Reed and Muench method[35]. The ten days LD_50_ for OB and OS in BALB/c mice with intranasal infections were 3.15x10^4^ CFU and 6.45x10^5^CFU, respectively. Based on the determined LD_50_, OS was ~20 times less virulent than OB.

### Intranasal Administration of Sub-Lethal Dose of *B*. *pseudomallei* Led to Persistent Infection in BALB/c Mice

**Fig 3:**
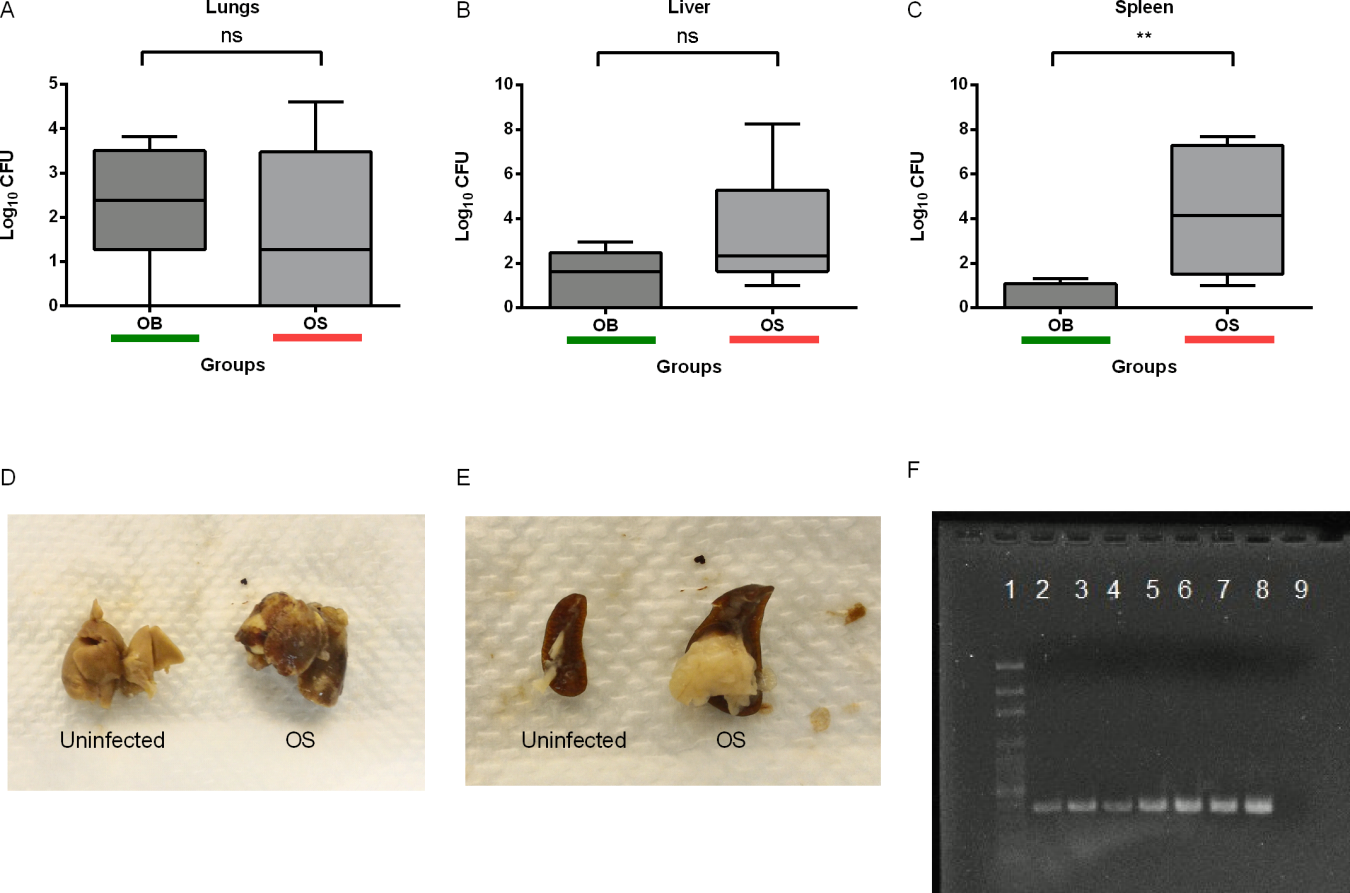
Persistent WT and SCV *B*. *pseudomallei* infection in BALB/c mice. Bacterial loads in (A) lungs, (B) livers, and (C) spleens harvested from each mouse after ≥60 days of sub-lethal dose of *B*. *pseudomallei* infection. Data are pooled from two independent experiments (A-C; n = 6 per group). (D) Liver and (E) splenic abscesses in OS-infected mice. Box plots show the median value (line), the interquartile range (box), and the minimum and maximum values (whisker). *P* values were calculated using Mann-Whitney U test. *P<0.05, **P<0.01, ***P<0.001. (F) The 397bp band on gel image indicates presence of *B*. *pseudomallei* in mice with persistent infection. Lane 1 shows DNA ladder. Lane 2–4 shows presence of OB in lung, liver, and spleen homogenate, respectively. Lane 5–7 shows presence of OS in lung, liver, and spleen homogenate, respectively. Lane 8–9 shows positive and negative control. Picture depicts one representative of all gel images.

We generated a persistent murine model to study host immune responses against persistent *B*. *pseudomallei* infection. A sublethal (~2–8% of the LD_50_) dose was used to inoculate groups of six mice via intranasal route. Persistent infection was defined as survival of mice despite *B*. *pseudomallei* infection for ≥60 days, and presence of the bacteria in lungs, livers or spleens[36]. Homogenates of lungs, livers or spleens harvested from mice with persistent *B*. *pseudomallei* infection after 60 days showed growth on selective Ashdown’s agar, with a detection limit of 20CFU/organ. Bacterial loads in lungs, livers, and spleens from each mouse were compared. We found that OS caused significantly higher bacterial burden in spleens than OB, but not in lungs and livers (Fig 3A–3C). In addition, we noted that OS was more likely to cause liver and splenic abscesses in mice compared to OB (Fig 3D and 3E). These data suggested that WT and SCV could have different pathogenesis *in vivo*. DNA was also extracted from colonies on Ashdown’s agar, and PCR which amplified the 397bp region corresponding to a conserved 16S rRNA sequence of *B*. *pseudomallei* was done to exclude the possibility of the presence of other bacteria species on Ashdown’s agar (Fig 3F).

### Persistent *B*. *pseudomallei* Infection Resulted in Significant Decrease of CD4+ T Cells and CD4+/CD8+ T Cell Ratios, but Increase in CD8+ T Cells

**Fig 4:**
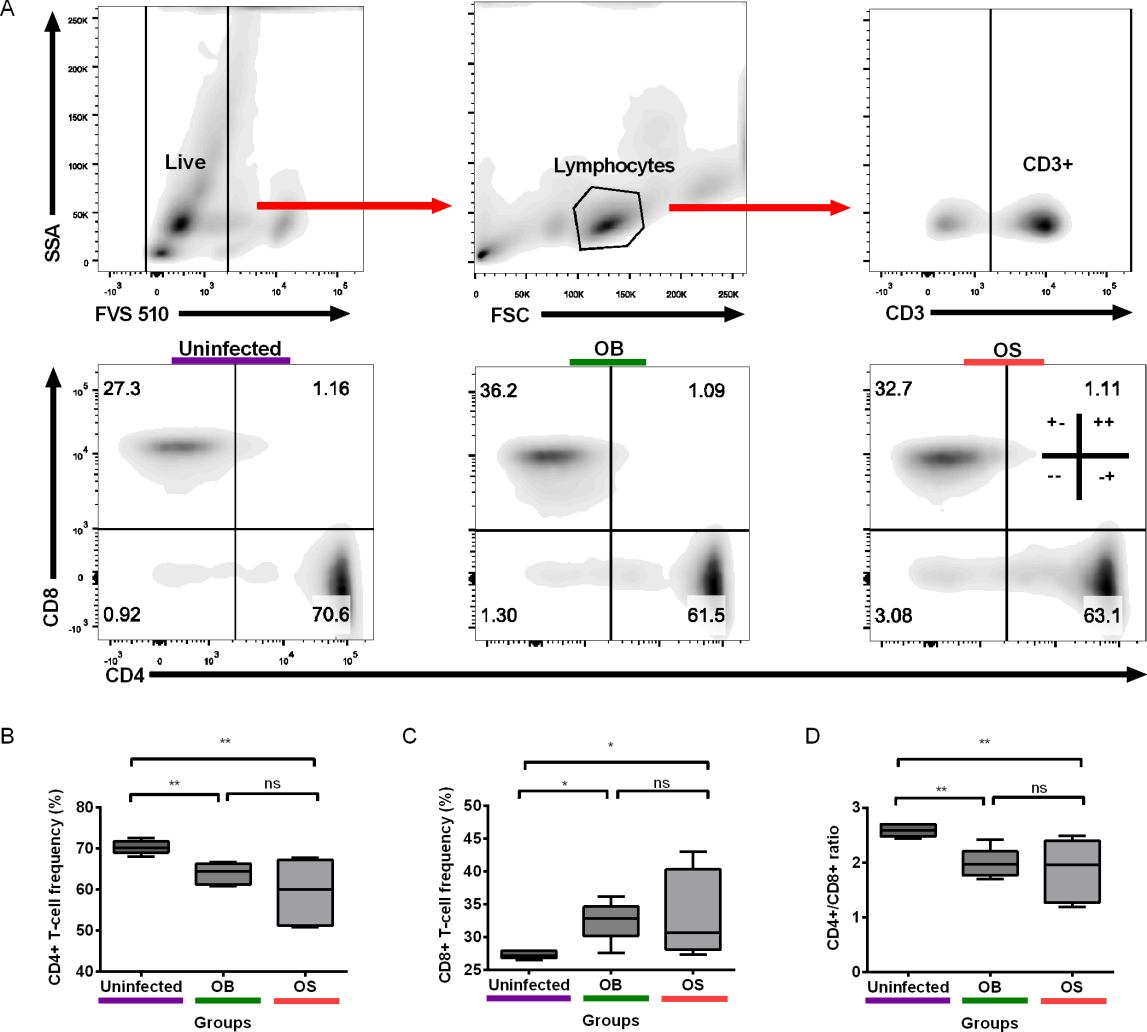
CD4+ and CD8+ T-cell frequencies in PBMCs. (A) Illustrations of the gating strategy used. All gates were set using respective isotype controls. One representative image from uninfected, OB-, and OS-infected mice is depicted. (B) CD4+ and (C) CD8+ T-cell frequencies and (D) CD4+/CD8+ T-cell ratios in PBMCs isolated from uninfected, OB- and OS-infected mice after 60 days of infection. Data representative of two independent experiments (B-D; n = 6 per group). Box plots show the median value (line), the interquartile range (box), and the minimum and maximum values (whiskers). *P* values were calculated using Mann-Whitney U test. *P<0.025, **P<0.005, ***P<0.0005 after Bonferroni correction for 2 comparisons.

We sought to compare the frequencies of CD4+ and CD8+ T cells between mice without and with persistent *B*. *pseudomallei* infection by using flow cytometry (Fig 4A). Our data revealed that the CD4+ T-cell frequencies were significantly decreased in both OB- and OS-infected than uninfected mice (Fig 4B). No statistical significance was seen between the two strains in their CD4+ T-cell frequencies. On the other hand, the CD8+ T-cell frequencies were remarkably increased in both OB- and OS-infected than uninfected mice. No statistical significance was observed for CD8+ T-cell frequencies between the two strains (Fig 4C). We also found that the CD4+/CD8+ T-cell ratios were remarkably reduced in the OB- and OS-infected than uninfected mice (Fig 4D). No statistical significance was found with CD4+/CD8+ ratios between the two strains. Together, we concluded that *B*. *pseudomallei* can alter both CD4+ and CD8+ T-cell frequencies during persistent infection, possibly suggesting that the ability of the bacteria to modulate host immune responses.

### Experimental Persistent Infection with *B*. *pseudomallei* Led to Upregulation of PD-1 on T Cells, but Not CTLA-4 in BALB/c Mice

**Fig 5:**
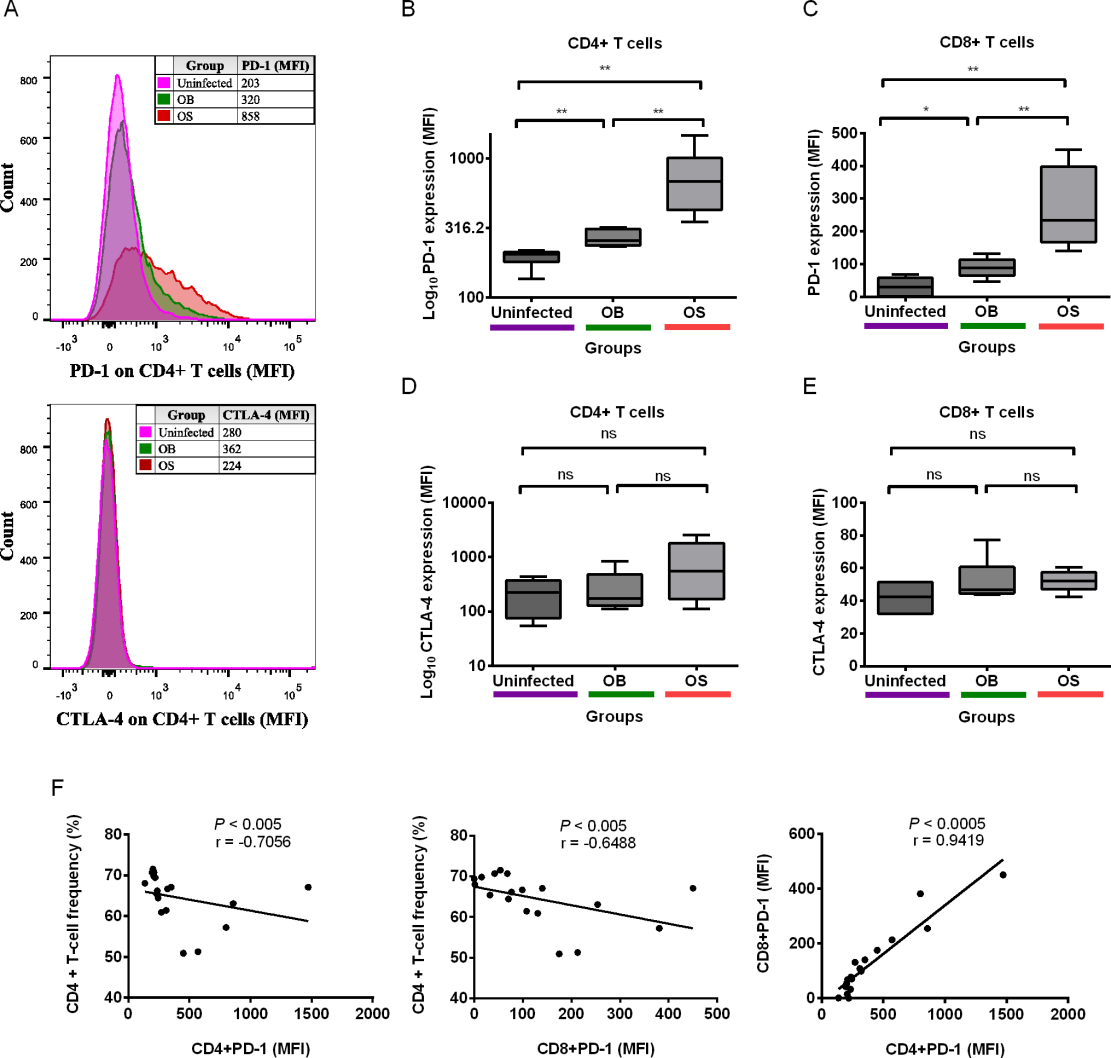
PD-1 and CTLA-4 expressions on FVS 510-/Lymph/CD3+/CD4+ and CD8+ T cells. (A) Representative histogram of PD-1 and CTLA-4 expressions on CD4+ T cells of uninfected, OB- and OS-infected mice after 60 days of infection. Median fluorescence intensities (MFIs) of PD-1 on (B) CD4+ and (C) CD8+ T cells, and (D) CTLA-4 on CD4+ and (E) CD8+ T cells of uninfected, OB- and OS-infected mice. Mean fluorescence intensity is used in (D) as median shows negative fluorescence values. Logarithm with base 10 is used for (B) and (D) for improved illustration purposes. Data are pooled from two independent experiments (B-E; n = 6 per group). Box plots show the median value (line), the interquartile range (box), and the minimum and maximum values (whiskers). Correlation analyses between CD4+ T-cell frequency and PD-1 expression on (F) CD4+ and CD8+ T cells, and between PD-1 expression on CD4+ and CD8+ T cells. Data are pooled from all uninfected, OB, and OS-infected mice from two independent experiments (F; total n = 18). *P* values calculated using Mann-Whitney U test (B–E) and Spearman’s Rank Order Coefficient (F). *P<0.025, **P<0.005, ***P<0.0005 after Bonferroni correction for 2 comparisons.

PD-1 and CTLA-4 belong to the CD28-B7 superfamily, and their expressions on T cells are reportedly upregulated in persistent infections such as tuberculosis and HIV [26–31]. Thus, we investigated if persistent *B*. *pseudomallei* infection led to any signs of immune exhaustion on T cells in mice. We found that PD-1 expression (MFI) was higher on CD4+ T cells of both OB- (median, 257; IQR, 75.8) and OS-infected (median, 686.0; IQR, 586.0), as compared to uninfected mice (median, 204.5; IQR, 32.7; Fig 5A and 5B). PD-1 expression was strain-dependent as we observed higher PD-1 MFI on CD4+ T cells of OS, as compared to OB-infected mice (*P*<0.005). At the same time, PD-1 MFI was significantly increased on both CD8+ T cells of OB- (median, 88.1; IQR, 49.3) and OS-infected (median, 233.5; IQR, 232) relative to uninfected mice (median, 28.9; IQR, 56.5; Fig 5C). We also found higher PD-1 expression on CD8+ T cells of OS, as compared to OB-infected mice (*P*<0.005).

Given the indispensable role of CD4+ T cells in mice with persistent *B*. *pseudomallei* infection[21], and that reduced CD4+ T-cell frequency and increased PD-1 expression on CD4+ and CD8+ T cells were observed in our experiments, it would be interesting to see if there exists any correlations between these parameters. We found significant negative correlations between CD4+ T-cell frequency and PD-1 expression on CD4+ T cells and also between CD4+ T-cell frequency and PD-1 expression on CD8+ T cells (Fig 5F). We also observed a strong positive correlation between PD-1 expression on CD4+ T cells and CD8+ T cells (Fig 5F). Besides PD-1, we also investigated CTLA-4 expressions on CD4+ and CD8+ T cells. Interestingly, we did not observe any statistical significance in CTLA-4 expression (MFI) on CD4+ T cells among OB-infected (median, 174.5; IQR, 349.3), OS-infected (median, 550.5; IQR, 1629.5) and uninfected mice (median, 222.5; IQR, 297.7; Fig 5A and 5D). We also found no significant differences in CTLA-4 expression on CD8+ T cells among OB-infected (median, 46.9; IQR, 16.1), OS-infected (median, 52.0; IQR, 10.3) and uninfected mice (median, 42.4; IQR, 19.3; Fig 5E). Together, our results suggest that PD-1 could be one of the inhibitory molecules that play an important role in persistent *B*. *pseudomallei* infection.

### Persistent Infection of BALB/c Mice with SCV *B*. *pseudomallei* Led to Pro-Inflammatory Plasma Cytokine Responses

**Fig 6:**
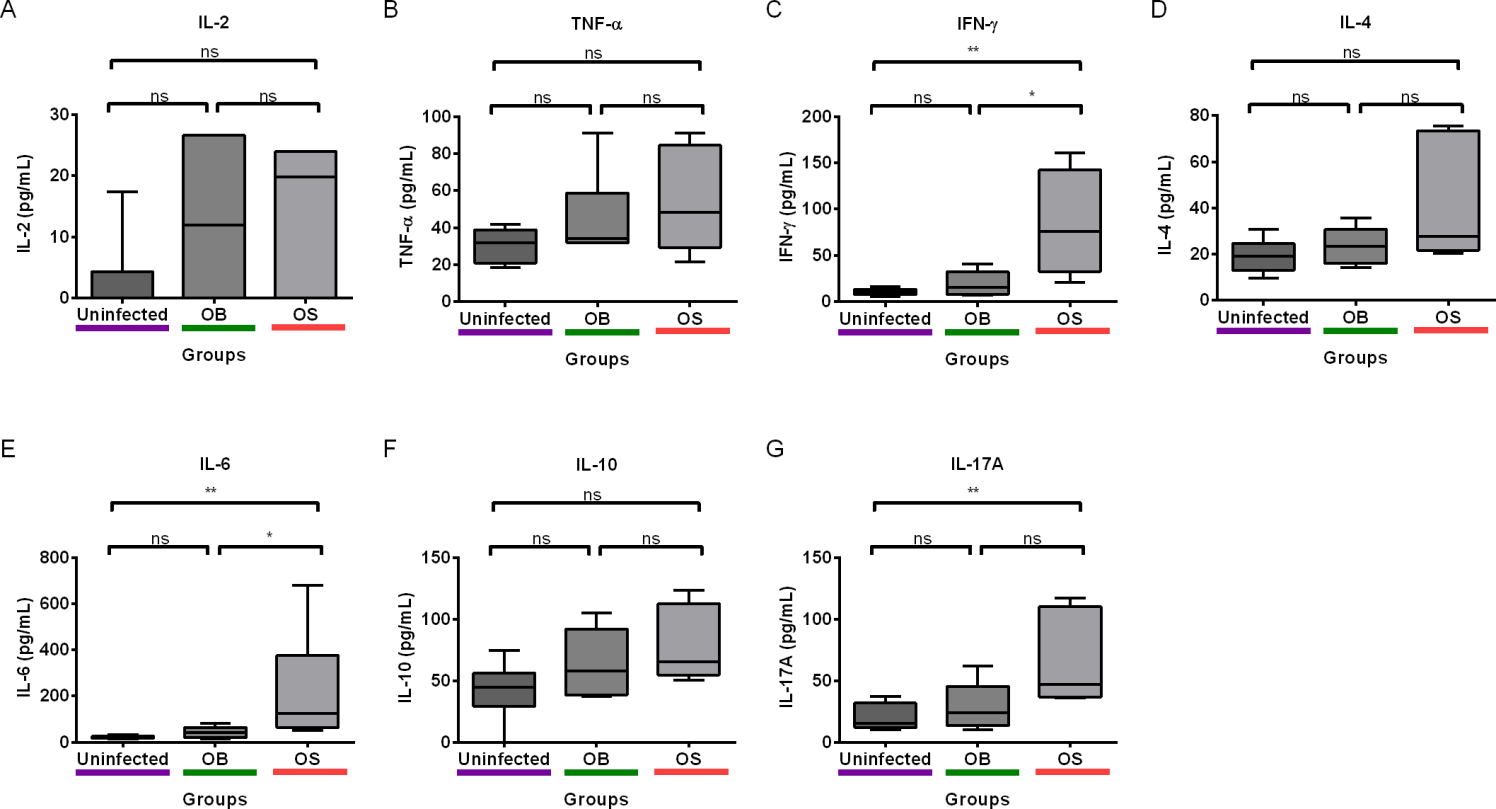
Levels of plasma Th1/Th2/Th17 cytokines in experimental persistent infection of *B*. *pseudomallei*. (A) IL-2 (B) TNF-α (C) IFN-γ (D) IL-4 and (E) IL-6 (F) IL-10 and (G) IL-17A levels were measured using flow cytometry. Data are pooled from two independent experiments (A–G; n = 6 per group). Box plots show the median value (line), the interquartile range (box), and the minimum and maximum values (whiskers). *P* values were calculated using Mann-Whitney U test. *P<0.025, **P<0.005, ***P<0.0005 after Bonferroni correction for 2 comparisons.

Since PD-1 negatively regulates T-cell functions, we asked whether PD-1 expressions on T cells in PBMCs from mice with persistent *B*. *pseudomallei* infection could alter plasma Th1, Th2, and Th17 cytokine levels. Cytokine levels were measured using a CBA mouse Th1/Th2/Th17 (BD Biosciences) assay by flow cytometry. Indeed, we found that several cytokines, especially pro-inflammatory cytokine levels in OS-infected mice were significantly higher than OB-infected and uninfected mice. IFN-γ and IL-6 levels in OS-infected mice were significantly higher than OB-infected and uninfected mice (Fig 6C and 6E). In addition, we observed higher IL-17A level in OS-infected as compared to uninfected mice, but not compared to OB-infected mice (Fig 6G). Interestingly, no significant difference in any cytokine level was found between OB-infected and uninfected mice. These results demonstrated that the SCV can trigger significantly the release of more pro-inflammatory IFN-γ, IL-6, and IL-17, possibly due to a higher bacterial load in spleens compared to WT (Fig 5C).

### Persistent Infection of BALB/c Mice with SCV *B*. *pseudomallei* Caused Skewed Th1 and Th17 Responses in Plasma Cytokines

**Fig 7:**
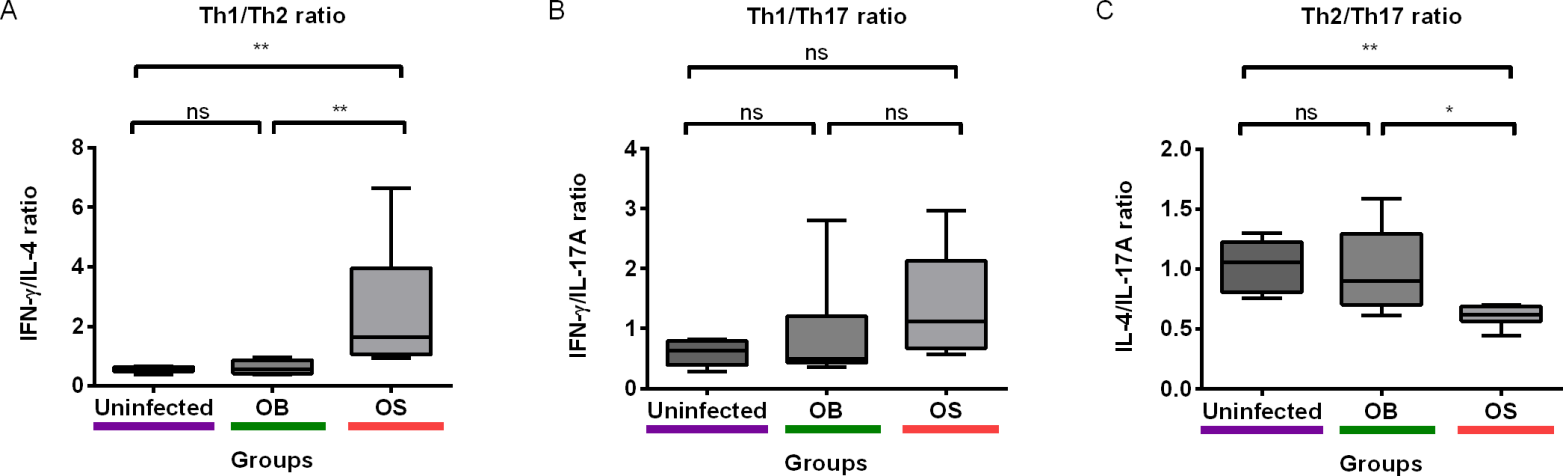
Plasma Th1/Th2/Th17 cytokine ratios. (A) Th1/Th2 (B) Th1/Th17 (C) Th2/Th17 ratios were measured in uninfected, OB- and OS-infected mice. Box plots show the median value (line), the interquartile range (box), and the minimum and maximum values (whiskers). *P* values were calculated using Mann-Whitney U test. *P<0.025, **P<0.005, ***P<0.0005 after Bonferroni correction for 2 comparisons.

Last but not the least, we asked if plasma cytokines were skewed toward Th1, Th2, or Th17 profiles in mice with persistent *B*. *pseudomallei* infection. We measured IFN-γ/IL-4, IFN-γ/IL-17A, and IL-4/IL-17A as an indication of Th1/Th2, Th1/Th17, and Th2/Th17 balance, respectively. We observed skewed Th1 and Th17 responses in OS-infected, as compared to OB-infected and uninfected mice (Fig 7A–7C). We found that Th1/Th17 ratio was higher, and Th2/Th17 ratio was lower in OS-infected relative to the OB-infected and uninfected mice. We further performed correlation analysis between the levels of plasma cytokines to better understand relationships between each cytokine (Table 1). Our investigations showed that IFN-γ positively correlated with IL-6, IL-10, and IL-17A. IL-6 correlated positively with IL-10 and IL-17A. IL-4 had a positive correlation with IL-10 and IL-17A. Lastly, IL-10 correlated positively with IL-17A. These results suggested a complex regulation between Th1, Th2, and Th17 cytokines in mice with persistent *B*. *pseudomallei* infection.

**Table 1:** Correlation analysis between Th1/Th2/Th17 cytokines. *P* values were calculated using Spearman’s Rank Order Coefficient. *P<0.008, **P<0.002, ***P<0.0002 after Bonferroni correction for 6 comparisons.

Cytokine	IL-2	TNF-α	IFN-γ	IL-4	IL-6	IL-10
**IL-2**						
**TNF-α**	ns					
**IFN-γ**	ns	ns				
**IL-4**	ns	ns	ns			
**IL-6**	ns	ns	r = 0.9427, ***P<0.0002	ns		
**IL-10**	ns	ns	r = 0.6139, *P<0.008	r = 0.9348, ***P<0.0002	r = 0.6366, *P<0.008	
**IL-17A**	ns	ns	r = 0.7703, **P = 0.0002	r = 0.9122, ***P<0.0002	r = 0.7909, ***P<0.0002	r = 0.9415, P***<0.0002

## Discussion

Two common mutants, *hemB* and *menD*, exhibit electron transport-defective SCVs phenotype [41,42]. There are mixed opinions in regard to the virulence of SCVs. Some have reported that the virulence of SCVs decreased in rabbit and *Caenorhabditis elegans* models [43–45]. A more recent data showed that clinical SCVs, *hemB*, *and menD* mutants of *S*. *aureus* were significantly less virulent than their genetically complemented isogenic strains [46]. Here, we showed that the SCV of *B*. *pseudomallei* is ~20-fold less virulent than its parental WT strain by determining the LD_50_ in a murine model. In contrast, Johnsson et al. have reported that NMRI mice infected with *hemB* mutant of *S*. *aureus* had a higher frequency and severity of arthritis than its isogenic parental strain[47]. The most important aspect to notice is Johnsson et al. have used severity of arthritis as the parameter to determine virulence of SCVs. We and others have used survival rate as the parameter [43–46]. The discrepancy in the measuring parameter for virulence might have produced different results. We propose that survival rate is a more accurate and objective parameter for determining the virulence of an organism, as severity of a disease could be subjective based on researchers and does not necessarily correlate to death. Thus, we believe that SCVs are less virulent, but more persistent in the host than their parental strains, which is congruent with the fact that SCVs have always been recovered from patients with chronic or relapsing persistent infections.

Persistent infections could possibly alter CD4+ and CD8+ T-cell frequencies, since these T cells are actively involved in cellular immune responses against infections. For instance, the hallmark of chronic HIV infections is CD4+ T-cell depletion, which occurs partly because HIV preferentially infects CD4+ T cells and triggers their apoptosis[48]. Our results revealed that CD4+ T-cell frequencies were lower in mice with persistent *B*. *pseudomallei* infection. These results might suggest the unique role of CD4+ T cells in conferring protection to mice with persistent *B*. *pseudomallei* infection, which was also demonstrated by the other study[21]. The mechanism behind the decrease in CD4+ T cells requires further investigation.

PD-1 causes T-cell exhaustions, and is reportedly upregulated in *Mycobacterium tuberculosis* (MTB) and HIV infections [26,28]. Nevertheless, the role of PD-1 in persistent *B*. *pseudomallei* infection has rarely been studied. Hence, we characterized the levels of PD-1+ T cells in persistent *B*. *pseudomallei* infection using a murine model, and compared between the SCV and its isogenic parental strain. We demonstrated for the first time that PD-1 expressions were increased on both CD4+ and CD8+ T cells in mice with persistent *B*. *pseudomallei* infection. Interestingly, mice infected with the SCV showed higher PD-1 expressions on both T cell subsets, as compared to its wild-type counterpart. There could be two possible explanations for this observation. The first explanation would be higher bacterial loads in spleens of SCV-infected mice led to higher inflammatory responses, thus a higher PD-1 expression on T-cells was required to limit inflammatory-induced pathology. Another explanation would be the distinct feature of SCVs in persistent infections. It is plausible that SCVs cause more persistent infections due to their relatively better abilities in impairing protective T-cell responses, rendering them better potential to persist in the host. Although PD-1 and CTLA-4 are closely related, our results showed that PD-1, but not CTLA-4, was one of the co-inhibitory molecules exploited by *B*. *pseudomallei* to possibly evade host immune responses, with SCVs having greater abilities in elevating PD-1 expression on T cells. Hence, it poses the possibility of using PD-1 pathway inhibitors against persistent *B*. *pseudomallei* infection; this also because PD-1 antagonists have shown remarkable results against different diseases particularly cancer [49]. Treatment with anti-PD-1/PD-L1 antibodies can reverse T-cell exhaustion and boost T-cell effector functions, which could bolster effective eradication of cancer cells and intracellular pathogens [49–51]. Several anti-PD-1/PD-L1 antibodies are commercially available, and many are currently in the pipe-line of evaluation in clinical trials [52]. Furthermore, an alternative treatment potentially with anti-PD-1/PD-L1 antibodies may be warranted in clinical melioidosis, as relapse can be as high as 10% despite appropriate antibiotic treatment[19]. In addition, immunotherapy with anti-PD-1/PD-L1 antibodies might also assist in eradicating the recalcitrant bacteria, thus preventing relapse of the disease.

Moreover, our data revealed that CD4+ T-cell frequency correlated inversely with PD-1 expression on CD4+ T cells and CD8+ T cells. Our study provides a clear association between PD-1 expression on CD4+ and CD8+ T cells and CD4+ T-cell frequency in persistent *B*. *pseudomallei* infection. We also observed ≥3-fold higher PD-1 expression on CD4+ than CD8+ T cells, suggesting distinct regulations in these T-cell subsets. We also found a strong positive correlation between PD-1 expressions on both CD4+ and CD8+ T cells, which indicate that persistent *B*. *pseudomallei* infection concertedly governs their expression T cell subsets. Our correlation results were in line with other HIV studies [28,53], which suggest that persistent *B*. *pseudomallei* infection can deplete the functional immune system.

Besides, we noted differential cytokine responses in only SCV-infected mice. Peripheral blood IFN-γ and IL-6 levels were significantly elevated in SCV-infected, compared to WT-infected and uninfected mice. Previous study on mice with chronic *B*. *pseudomallei* infection showed that IFN-γ and IL-6 levels in sera were only increased in bacteremic mice, compared to non-bacteremic mice[54]. Indeed, we observed that survival mice infected with the SCV after two months were more likely to develop liver or splenic abscesses compared to the WT. We did not observe significant changes in lungs, despite using intranasal route for infection. Our observation can be supported by previous persistent model study that demonstrated higher bacterial recovery percentage from livers and spleens compared to lungs after intranasal challenge[36]. We speculate that the SCV is the one that is more likely to cause severe persistent disease, which was indicated by the higher bacterial loads in spleens compared to the WT. This possibly contributed to IFN-γ and IL-6 upregulation. We think that mice infected with the WT would either succumb to the disease within two months due to higher virulence of the WT, or their immunity are able to control the bacterial burden to very minimal amount, which will not trigger massive cytokine responses. We also found a positive correlation between IFN-γ and IL-6 levels, which shows that they were synergistically released in response to the disease. Interestingly, we also observed higher plasma IL-17A level in SCV-infected mice compared to uninfected, but not compared to WT-infected mice. IL-17A is a pro-inflammatory Th17 signature cytokine that controls bacterial infections by recruiting neutrophils [55]. IL-17A could have been released more in the SCV-infected mice as part of the inflammatory process, which however needs to be investigated further.

Our results showed a skewed Th1 and Th17 responses in the SCV-infected mice, as compared to the WT-infected and uninfected mice. SCVs are known to persist intracellularly better than the WT, leading to persistent infections [56]. This explains the skewed Th1 responses in the SCV-infected mice, and that Th1 cells are mainly responsible for eradicating intracellular pathogens [57]. The role of Th17 responses against intracellular pathogens are seldom well understood, but few pieces of evidence have demonstrated that Th17 responses promote Th1 immunity to pulmonary intracellular bacterial infections [58,59]. Our results suggest the possibility of skewed Th17 responses that are required for mediating Th1 responses against intracellular SCVs of *B*. *pseudomallei*, which warrants further studies. We acknowledge our study limitation in addressing changes of bacterial loads, cytokine levels, and PD-1 expression over a time course, which can provide more useful insights to understand immunopathogenesis caused by the SCV and the WT. Despite that, we demonstrated for the first time that the SCV was different from the WT in the aspect of virulence, bacterial loads, PD-1 expression, and cytokine level in mice with persistent *B*. *pseudomallei* infection. Future studies are warranted preferably using blocking antibodies or gene-knock-out murine models to examine the direct effects of eliminating co-inhibitory or cytokine genes in regulating persistent SCV and WT *B*. *pseudomallei* infection over a time course.

## References

[1] White NJ (2003) Melioidosis. Lancet 361: 1715–1722. 12767750 10.1016/s0140-6736(03)13374-0

[2] Sprague LD, Neubauer H (2004) Melioidosis in animals: a review on epizootiology, diagnosis and clinical presentation. J Vet Med B Infect Dis Vet Public Health 51: 305–320. 15525357 10.1111/j.1439-0450.2004.00797.x

[3] Wiersinga WJ, Currie BJ, Peacock SJ (2012) Melioidosis. N Engl J Med 367: 1035–1044. 10.1056/NEJMra1204699 22970946

[4] Koponen MA, Zlock D, Palmer DL, Merlin TL (1991) Melioidosis. Forgotten, but not gone! Arch Intern Med 151: 605–608. 2001144 10.1001/archinte.151.3.605

[5] Mays EE, Ricketts EA (1975) Melioidosis: recrudescence associated with bronchogenic carcinoma twenty-six years following initial geographic exposure. Chest 68: 261–263. 1149556 10.1378/chest.68.2.261

[6] Ngauy V, Lemeshev Y, Sadkowski L, Crawford G (2005) Cutaneous melioidosis in a man who was taken as a prisoner of war by the Japanese during World War II. J Clin Microbiol 43: 970–972. 15695721 10.1128/JCM.43.2.970-972.2005 PMC548040

[7] Kahl B, Herrmann M, Everding AS, Koch HG, Becker K, et al (1998) Persistent infection with small colony variant strains of Staphylococcus aureus in patients with cystic fibrosis. J Infect Dis 177: 1023–1029. 9534977 10.1086/515238

[8] Proctor RA, van Langevelde P, Kristjansson M, Maslow JN, Arbeit RD (1995) Persistent and relapsing infections associated with small-colony variants of Staphylococcus aureus. Clin Infect Dis 20: 95–102. 7727677 10.1093/clinids/20.1.95

[9] von Eiff C, Bettin D, Proctor RA, Rolauffs B, Lindner N, et al (1997) Recovery of small colony variants of Staphylococcus aureus following gentamicin bead placement for osteomyelitis. Clin Infect Dis 25: 1250–1251. 9402396 10.1086/516962

[10] Spanu T, Romano L, D'Inzeo T, Masucci L, Albanese A, et al (2005) Recurrent ventriculoperitoneal shunt infection caused by small-colony variants of Staphylococcus aureus. Clin Infect Dis 41: e48–52. 16080075 10.1086/432577

[11] Abele-Horn M, Schupfner B, Emmerling P, Waldner H, Goring H (2000) Persistent wound infection after herniotomy associated with small-colony variants of Staphylococcus aureus. Infection 28: 53–54. 10697795 10.1007/s150100050014

[12] Quie PG (1969) Microcolonies (G-variants) of Staphylococcus aureus. Yale J Biol Med 41: 394–403. 5780692 PMC2591491

[13] Wise RI (1956) Small colonies (G variants) of staphylococci: isolation from cultures and infections. Ann N Y Acad Sci 65: 169–174. 13363212 10.1111/j.1749-6632.1956.tb36636.x

[14] Proctor RA, von Eiff C, Kahl BC, Becker K, McNamara P, et al (2006) Small colony variants: a pathogenic form of bacteria that facilitates persistent and recurrent infections. Nat Rev Microbiol 4: 295–305. 16541137 10.1038/nrmicro1384

[15] Wise RI, Spink WW (1954) The influence of antibiotics on the origin of small colonies (G variants) of Micrococcus pyogenes var. aureus. J Clin Invest 33: 1611–1622. 13211817 10.1172/JCI103041 PMC1072592

[16] Haussler S, Rohde M, Steinmetz I (1999) Highly resistant Burkholderia pseudomallei small colony variants isolated in vitro and in experimental melioidosis. Med Microbiol Immunol 188: 91–97. 10753061 10.1007/s004300050110

[17] Al-Maleki AR, Mariappan V, Vellasamy KM, Shankar EM, Tay ST, et al (2014) Enhanced intracellular survival and epithelial cell adherence abilities of Burkholderia pseudomallei morphotypes are dependent on differential expression of virulence-associated proteins during mid-logarithmic growth phase. J Proteomics 106: 205–220. 10.1016/j.jprot.2014.04.005 24742602

[18] Al-Maleki AR, Mariappan V, Vellasamy KM, Tay ST, Vadivelu J (2015) Altered Proteome of Burkholderia pseudomallei Colony Variants Induced by Exposure to Human Lung Epithelial Cells. PLoS One 10: e0127398 10.1371/journal.pone.0127398 25996927 PMC4440636

[19] Limmathurotsakul D, Chaowagul W, Chierakul W, Stepniewska K, Maharjan B, et al (2006) Risk factors for recurrent melioidosis in northeast Thailand. Clin Infect Dis 43: 979–986. 16983608 10.1086/507632

[20] Jenjaroen K, Chumseng S, Sumonwiriya M, Ariyaprasert P, Chantratita N, et al (2015) T-Cell Responses Are Associated with Survival in Acute Melioidosis Patients. PLoS Negl Trop Dis 9: e0004152 10.1371/journal.pntd.0004152 26495852 PMC4619742

[21] Haque A, Easton A, Smith D, O'Garra A, Van Rooijen N, et al (2006) Role of T cells in innate and adaptive immunity against murine Burkholderia pseudomallei infection. J Infect Dis 193: 370–379. 16388484 10.1086/498983

[22] Freeman GJ, Long AJ, Iwai Y, Bourque K, Chernova T, et al (2000) Engagement of the PD-1 immunoinhibitory receptor by a novel B7 family member leads to negative regulation of lymphocyte activation. J Exp Med 192: 1027–1034. 11015443 10.1084/jem.192.7.1027 PMC2193311

[23] Latchman Y, Wood CR, Chernova T, Chaudhary D, Borde M, et al (2001) PD-L2 is a second ligand for PD-1 and inhibits T cell activation. Nat Immunol 2: 261–268. 11224527 10.1038/85330

[24] Linsley PS, Brady W, Urnes M, Grosmaire LS, Damle NK, et al (1991) CTLA-4 is a second receptor for the B cell activation antigen B7. J Exp Med 174: 561–569. 1714933 10.1084/jem.174.3.561 PMC2118936

[25] Freeman GJ, Gribben JG, Boussiotis VA, Ng JW, Restivo VA Jr., et al (1993) Cloning of B7-2: a CTLA-4 counter-receptor that costimulates human T cell proliferation. Science 262: 909–911. 7694363 10.1126/science.7694363

[26] Singh A, Mohan A, Dey AB, Mitra DK (2013) Inhibiting the programmed death 1 pathway rescues Mycobacterium tuberculosis-specific interferon gamma-producing T cells from apoptosis in patients with pulmonary tuberculosis. J Infect Dis 208: 603–615. 10.1093/infdis/jit206 23661793

[27] Schreiber HA, Hulseberg PD, Lee J, Prechl J, Barta P, et al (2010) Dendritic cells in chronic mycobacterial granulomas restrict local anti-bacterial T cell response in a murine model. PLoS One 5: e11453 10.1371/journal.pone.0011453 20625513 PMC2897891

[28] Day CL, Kaufmann DE, Kiepiela P, Brown JA, Moodley ES, et al (2006) PD-1 expression on HIV-specific T cells is associated with T-cell exhaustion and disease progression. Nature 443: 350–354. 16921384 10.1038/nature05115

[29] Kaufmann DE, Kavanagh DG, Pereyra F, Zaunders JJ, Mackey EW, et al (2007) Upregulation of CTLA-4 by HIV-specific CD4+ T cells correlates with disease progression and defines a reversible immune dysfunction. Nat Immunol 8: 1246–1254. 17906628 10.1038/ni1515

[30] Kirman J, McCoy K, Hook S, Prout M, Delahunt B, et al (1999) CTLA-4 blockade enhances the immune response induced by mycobacterial infection but does not lead to increased protection. Infect Immun 67: 3786–3792. 10417139 10.1128/iai.67.8.3786-3792.1999 PMC96655

[31] Jurado JO, Alvarez IB, Pasquinelli V, Martinez GJ, Quiroga MF, et al (2008) Programmed death (PD)-1:PD-ligand 1/PD-ligand 2 pathway inhibits T cell effector functions during human tuberculosis. J Immunol 181: 116–125. 18566376 10.4049/jimmunol.181.1.116

[32] Buddhisa S, Rinchai D, Ato M, Bancroft GJ, Lertmemongkolchai G (2015) Programmed death ligand 1 on Burkholderia pseudomallei-infected human polymorphonuclear neutrophils impairs T cell functions. J Immunol 194: 4413–4421. 10.4049/jimmunol.1402417 25801435

[33] Ramli NS, Eng Guan C, Nathan S, Vadivelu J (2012) The effect of environmental conditions on biofilm formation of Burkholderia pseudomallei clinical isolates. PLoS One 7: e44104 10.1371/journal.pone.0044104 22970167 PMC3435415

[34] Suppiah J, Thimma JS, Cheah SH, Vadivelu J (2010) Development and evaluation of polymerase chain reaction assay to detect Burkholderia genus and to differentiate the species in clinical specimens. FEMS Microbiol Lett 306: 9–14. 10.1111/j.1574-6968.2010.01923.x 20345378

[35] Reed LJ, Muench H (1938) A simple method of estimating fifty per cent endpoints. The American Journal of Hygiene 27: 493–497.

[36] Goodyear A, Bielefeldt-Ohmann H, Schweizer H, Dow S (2012) Persistent gastric colonization with Burkholderia pseudomallei and dissemination from the gastrointestinal tract following mucosal inoculation of mice. PLoS One 7: e37324 10.1371/journal.pone.0037324 22624016 PMC3356274

[37] Shankar EM, Che KF, Messmer D, Lifson JD, Larsson M (2011) Expression of a broad array of negative costimulatory molecules and Blimp-1 in T cells following priming by HIV-1 pulsed dendritic cells. Mol Med 17: 229–240. 10.2119/molmed.2010.00175 21103670 PMC3060986

[38] Che KF, Shankar EM, Muthu S, Zandi S, Sigvardsson M, et al (2012) p38 Mitogen-activated protein kinase/signal transducer and activator of transcription-3 pathway signaling regulates expression of inhibitory molecules in T cells activated by HIV-1-exposed dendritic cells. Mol Med 18: 1169–1182. 10.2119/molmed.2012.00103 22777388 PMC3510300

[39] Ashdown LR (1979) An improved screening technique for isolation of Pseudomonas pseudomallei from clinical specimens. Pathology 11: 293–297. 460953 10.3109/00313027909061954

[40] Dharakul T, Songsivilai S, Viriyachitra S, Luangwedchakarn V, Tassaneetritap B, et al (1996) Detection of Burkholderia pseudomallei DNA in patients with septicemic melioidosis. J Clin Microbiol 34: 609–614. 8904424 10.1128/jcm.34.3.609-614.1996 PMC228856

[41] von Eiff C, Heilmann C, Proctor RA, Woltz C, Peters G, et al (1997) A site-directed Staphylococcus aureus hemB mutant is a small-colony variant which persists intracellularly. J Bacteriol 179: 4706–4712. 9244256 10.1128/jb.179.15.4706-4712.1997 PMC179315

[42] Bates DM, von Eiff C, McNamara PJ, Peters G, Yeaman MR, et al (2003) Staphylococcus aureus menD and hemB mutants are as infective as the parent strains, but the menadione biosynthetic mutant persists within the kidney. J Infect Dis 187: 1654–1661. 12721946 10.1086/374642

[43] Miller MH, Wexler MA, Steigbigel NH (1978) Single and combination antibiotic therapy of Staphylococcus aureus experimental endocarditis: emergence of gentamicin-resistant mutants. Antimicrob Agents Chemother 14: 336–343. 251069 10.1128/aac.14.3.336 PMC352461

[44] Pelletier LL Jr., Richardson M, Feist M (1979) Virulent gentamicin-induced small colony variants of Staphylococcus aureus. J Lab Clin Med 94: 324–334. 458250

[45] Sifri CD, Baresch-Bernal A, Calderwood SB, von Eiff C (2006) Virulence of Staphylococcus aureus small colony variants in the Caenorhabditis elegans infection model. Infect Immun 74: 1091–1096. 16428756 10.1128/IAI.74.2.1091-1096.2006 PMC1360298

[46] Dean MA, Olsen RJ, Long SW, Rosato AE, Musser JM (2014) Identification of point mutations in clinical Staphylococcus aureus strains that produce small-colony variants auxotrophic for menadione. Infect Immun 82: 1600–1605. 10.1128/IAI.01487-13 24452687 PMC3993378

[47] Jonsson IM, von Eiff C, Proctor RA, Peters G, Ryden C, et al (2003) Virulence of a hemB mutant displaying the phenotype of a Staphylococcus aureus small colony variant in a murine model of septic arthritis. Microb Pathog 34: 73–79. 12623275 10.1016/s0882-4010(02)00208-5

[48] Douek DC, Brenchley JM, Betts MR, Ambrozak DR, Hill BJ, et al (2002) HIV preferentially infects HIV-specific CD4+ T cells. Nature 417: 95–98. 11986671 10.1038/417095a

[49] Pauken KE, Wherry EJ (2015) Overcoming T cell exhaustion in infection and cancer. Trends Immunol 36: 265–276. 10.1016/j.it.2015.02.008 25797516 PMC4393798

[50] Larsson M, Shankar EM, Che KF, Saeidi A, Ellegard R, et al (2013) Molecular signatures of T-cell inhibition in HIV-1 infection. Retrovirology 10: 31 10.1186/1742-4690-10-31 23514593 PMC3610157

[51] Velu V, Shetty RD, Larsson M, Shankar EM (2015) Role of PD-1 co-inhibitory pathway in HIV infection and potential therapeutic options. Retrovirology 12: 14 10.1186/s12977-015-0144-x 25756928 PMC4340294

[52] Swaika A, Hammond WA, Joseph RW (2015) Current state of anti-PD-L1 and anti-PD-1 agents in cancer therapy. Mol Immunol 67: 4–17. 10.1016/j.molimm.2015.02.009 25749122

[53] D'Souza M, Fontenot AP, Mack DG, Lozupone C, Dillon S, et al (2007) Programmed death 1 expression on HIV-specific CD4+ T cells is driven by viral replication and associated with T cell dysfunction. J Immunol 179: 1979–1987. 17641065 10.4049/jimmunol.179.3.1979

[54] Conejero L, Patel N, de Reynal M, Oberdorf S, Prior J, et al (2011) Low-dose exposure of C57BL/6 mice to burkholderia pseudomallei mimics chronic human melioidosis. Am J Pathol 179: 270–280. 10.1016/j.ajpath.2011.03.031 21703409 PMC3123849

[55] Ye P, Garvey PB, Zhang P, Nelson S, Bagby G, et al (2001) Interleukin-17 and lung host defense against Klebsiella pneumoniae infection. Am J Respir Cell Mol Biol 25: 335–340. 11588011 10.1165/ajrcmb.25.3.4424

[56] Proctor RA, Kriegeskorte A, Kahl BC, Becker K, Loffler B, et al (2014) Staphylococcus aureus Small Colony Variants (SCVs): a road map for the metabolic pathways involved in persistent infections. Front Cell Infect Microbiol 4: 99 10.3389/fcimb.2014.00099 25120957 PMC4112797

[57] Kidd P (2003) Th1/Th2 balance: the hypothesis, its limitations, and implications for health and disease. Altern Med Rev 8: 223–246. 12946237

[58] Lin Y, Ritchea S, Logar A, Slight S, Messmer M, et al (2009) IL-17 is required for Th1 immunity and host resistance to the intracellular pathogen Francisella tularensis LVS. Immunity 31: 799–810. 10.1016/j.immuni.2009.08.025 19853481 PMC2789998

[59] Bai H, Cheng J, Gao X, Joyee AG, Fan Y, et al (2009) IL-17/Th17 promotes type 1 T cell immunity against pulmonary intracellular bacterial infection through modulating dendritic cell function. J Immunol 183: 5886–5895. 10.4049/jimmunol.0901584 19812198

